# The impact of point defects in n-type GaN layers on thermal decomposition of InGaN/GaN QWs

**DOI:** 10.1038/s41598-021-81017-w

**Published:** 2021-01-28

**Authors:** Mikolaj Grabowski, Ewa Grzanka, Szymon Grzanka, Artur Lachowski, Julita Smalc-Koziorowska, Robert Czernecki, Roman Hrytsak, Joanna Moneta, Grzegorz Gawlik, Andrzej Turos, Mike Leszczyński

**Affiliations:** 1 Institute of High Pressure Physics, Polish Academy of Sciences, Sokolowska 29/37, 01-142 Warsaw, Poland; 2 Top-GaN Ltd, Solec 24/90, 00-403 Warsaw, Poland; 3 College of Natural Sciences, Institute of Physics, University of Rzeszow, Pigonia 1, 35-959 Rzeszow, Poland; 4 Łukasiewicz Research Network - Institute of Microelectronics and Photonics, al. Lotników 32/46, 02-668 Warsaw, Poland; 5 National Centre for Nuclear Research, Andrzeja Soltana 7, 05-400 Otwock, Poland

**Keywords:** Condensed-matter physics, Materials for devices

## Abstract

The aim of this paper is to give an experimental evidence that point defects (most probably gallium vacancies) induce decomposition of InGaN quantum wells (QWs) at high temperatures. In the experiment performed, we implanted GaN:Si/sapphire substrates with helium ions in order to introduce a high density of point defects. Then, we grew InGaN QWs on such substrates at temperature of 730 °C, what caused elimination of most (but not all) of the implantation-induced point defects expanding the crystal lattice. The InGaN QWs were almost identical to those grown on unimplanted GaN substrates. In the next step of the experiment, we annealed samples grown on unimplanted and implanted GaN at temperatures of 900 °C, 920 °C and 940 °C for half an hour. The samples were examined using Photoluminescence, X-ray Diffraction and Transmission Electron Microscopy. We found out that the decomposition of InGaN QWs started at lower temperatures for the samples grown on the implanted GaN substrates what provides a strong experimental support that point defects play important role in InGaN decomposition at high temperatures.

## Introduction

InGaN/GaN quantum wells (QWs) are used in blue (wavelength emission: λ > 450 nm) and green (λ  > 500 nm) Light Emitting Diodes (LEDs) and Laser Diodes (LDs) manufactured for different application like lighting, displays, communication, as well as in the niche markets of Quantum Technologies, medicine or environmental protection. However, the technology of InGaN is far from being mature and there are still some properties of this material not well understood. InGaN quantum wells are grown at relatively low temperatures. For higher In content in green emitters, it is of only 700 °C. Additionally, InGaN has a large lattice mismatch to GaN: 10% between GaN and InN. Those two factors cause a high defect density in InGaN layers: In-content fluctuations^1^, high concentration of point defects^2^ and misfit dislocations for the layers above the critical thickness^3^. Such defects contribute to so called “green gap” i.e., low luminousity of the emitters in the green region of the spectrum. Last few years problem of point defects migration during the growth from n-type layers towards QWs and their influence on optical properties is widely discussed^4,5^.

Additional problem is an InGaN decomposition in active region during high temperature growth of p-type GaN and AlGaN layers above the QWs. This problem has been reported by a number of researchers^6–8^. In our research to date, we have observed that the decomposition of the multi QWs stack usually begins at the deepest, i.e., first grown QW. As there is no significant structural difference between the first and other quantum wells, the interaction between the InGaN QWs and the layers below should play a role. Since Si-doped n-type GaN layer grown in MOVPE method (under the N-rich conditions) contains a high concentration of Ga-vacancies^9^ we have posed a hypothesis that point defects migration could be responsible for the InGaN QWs decomposition at high temperatures.

In order to check this hypothesis, we have performed the experiment consisting of three steps: (i) implantation of GaN:Si layer grown on sapphire template with He-ions to increase significantly the point defect concentration^10^, (ii) growth of five-fold InGaN/GaN QWs on the unimplanted and implanted GaN layers at temperatures 730 °C—QWs and 810 °C—quantum barriers (QBs), (iii) annealing of these samples at temperatures 900 °C, 920 °C and 940 °C.

Experiments described in Refs.^11,12^ on annealing of implanted layers at temperatures above 600 °C showed that the structural damage produced by light ion bombardment at low fluences recovers completely, whereas after implantation with high mass ions and/or high fluence the recovery is only partial. That is why He-ions were chosen for implantation in our experiment. Such light atoms mainly introduce simple defects, i.e., defects having low energy formation and migration barrier, e.g., point defects as vacancies, interstitials atoms and also Frenkel pairs and antisite atoms, which have been observed as non-radiative recombination centers^13–15^. Additionally, since our experiment was focused on elucidation of the role of point defects migration on the decomposition of QWs, ion implantation conditions (ion energy and fluence) have been set to avoid formation of extended defects and consequently plastic deformation in the implanted sample^16^.

The characterization of the implanted GaN layers, using Photoluminescence (PL) and Cathodoluminescence (CL) methods showed that even a small amount of ions cause signal quenching^11,17^. In addition, CL spectra of as-implanted GaN is quenched up to depth beyond the ion penetration range, calculated by TRIM^18^. It can be attributed to the diffusion of simple defects created during ion-implantation. This indicates their high mobility over wide range of temperatures^11,13^.

Our paper is arranged in three parts. In the first one, the impact of the implantation on the GaN:Si layer will be discussed. In the second one, the results for the InGaN/GaN QWs grown on the He-implanted and unimplanted GaN:Si layers will be shown. In the third part, the results of annealing of InGaN/GaN multi QWs will be given and discussed.

## Experiment

GaN layer was grown using MOVPE (Metalorganic Chemical Vapour Phase Epitaxy)—AIXTRON CCS 3 × 2″ reactor. The sample was grown on sapphire in a sequence: 0.1 μm of LT (low temperature) GaN nucleation layer, 1.5 μm of undoped GaN at 300 mbar and 1050 °C and 1 μm of GaN:Si (Si concentration of 2.7 × 10^18^ cm^−3^) at 150 mbar and 1000 °C. Trimethylgallium (TMGa) was used as Ga precursors, ammonia as nitrogen source and H_2_ as a carrier gas. Silicon doping was realized using SiH_4_.

Such sample was divided into two parts: one of them was implanted with He-ions at room temperature (RT), second one, remained unimplanted. In order to obtain uniform implantation defect distribution He-ions were implanted with three energies: E_1_ = 25 keV, E_2_ = 75 keV, E_3_ = 160 keV and three different fluences: I_1_ = 1.9 × 10^14^ cm^−2^, I_2_ = 2.7 × 10^14^ cm^−2^, I_3_ = 5.0 × 10^14^ cm^−2^, respectively. Before implantation, GaN:Si layer was covered at low temperature by plasma-enhanced chemical vapor deposition (PECVD) with 20 nm thick SiN layer to avoid surface contamination. This layer was removed after implantation.

**Figure 1:**
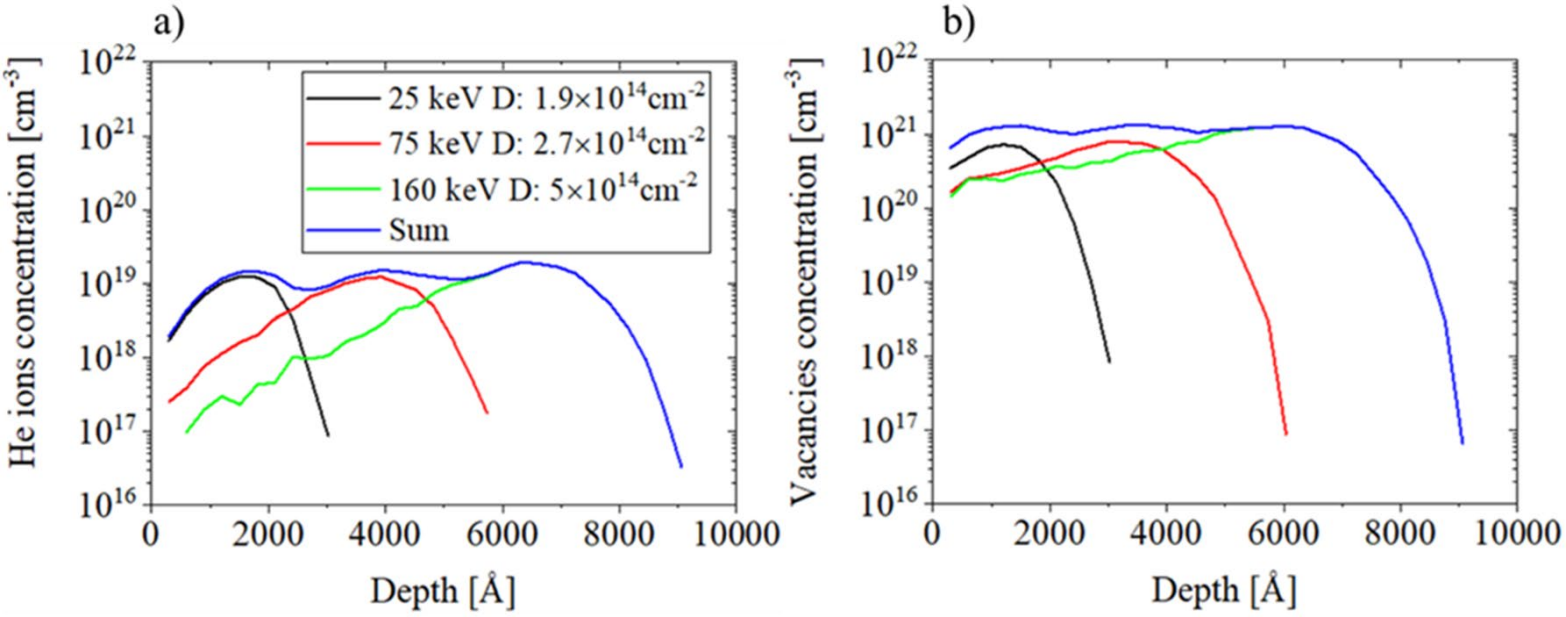
TRIM simulation of the depth profile of (**a**) implanted He ions concentration and (**b**) vacancies concentration in the GaN layer with 20 nm SiN cap on the top after multiple-step He ion implantation. The legend is the same for both figures.

Figure 1 presents TRIM simulations of impurity and defect profiles. One can see that ion implantation with three fluences and three energies of the He ions give quasi-uniform depth distribution of the vacancies. The surface morphology of both samples was investigated using Atomic Force Microscopy (AFM), using NanoScope Dimension 3100 in tapping mode with standard Si tip. High Resolution X-Ray Diffraction (HRXRD) and Scanning Transmission Electron Microscopy (STEM) methods were used for structural sample characterization. Measurements of HRXRD were performed using Empyrean (Malvern Panalytical) X-ray diffractometer operating at the CuKα_1_ wavelength, equipped with the hybrid 2bounce monochromator and a threefold Ge (220) analyzer. For each sample, 2Θ/ω scan of (0002) symmetrical reflection was measured and lattice parameters change was calculated. STEM images were taken using a FEI TECNAI G2 F20 S-TWIN microscope operating at 200 kV. The cross-sectional STEM specimens were prepared in a conventional way by using mechanical polishing followed by argon ion-milling at 4 keV. The optical properties of the samples were determined using photoluminescence (PL). Spectra were measured at room temperature (RT) and in a liquid nitrogen cryostat, excitation was obtained by an 325 nm He-CD laser, worked at continuous-wave operation mode. PL was recorded using Horiba iHR 320 Imaging Spectrometer and CCD camera as a detector.

**Figure 2:**
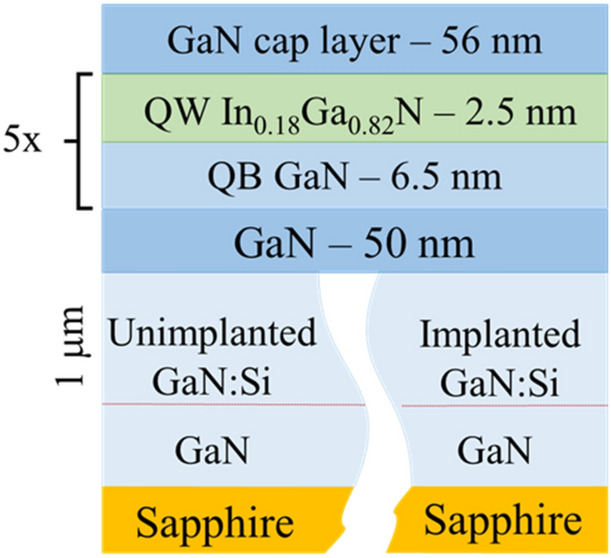
The scheme of InGaN/GaN MQWs grown on layers described in paragraph GaN:Si layers: unimplanted and implanted.

In the next step, five InGaN/GaN QWs were grown using MOVPE method, on unimplanted and implanted GaN:Si layer. Before regrowth, both samples, were annealed for 90 s at 950 °C and 150 mbar in NH_3_ atmosphere in the MOVPE reactor_._ After that, a 50 nm thick GaN layer was grown at 950 °C and 150 mbar with N/III ratio of 2000. Then, five nominally 2.5 nm thick InGaN QWs with 18% of indium content and 6.5 nm thick GaN QBs, were grown at 730 °C and 810 °C, respectively. These multi QWs/QBs were covered with a 56 nm GaN cap layer grown at 850˚C. Triethylgallium (TEGa) and trimethylindium (TMIn) were used as Ga and In precursors, ammonia as nitrogen source. QBs were grown using H_2_ as a carrier gas, whereas QWs were grown using N_2_. The epi-structure is schematically shown in Fig. 2.

Finally, the InGaN/GaN samples grown on unimplanted and implanted GaN:Si layers were divided into four pieces. Six samples (three pieces of MQWs grown on unimplanted layer and three pieces of MQWs grown on implanted layer) were annealed at temperatures of 900 °C, 920 °C and 940 °C in NH_3_ + H_2_ atmosphere, for 30 min in the MOVPE reactor.

All annealed samples and as grown MQWs samples were once more characterized using AFM, STEM, HRXRD and PL. Additionally, using Epitaxy software (Malvern Panalytical) it was possible to calculate theoretical diffraction pattern. The calculated patterns were fitted to the measured data, allowing determination of QWs and QBs thickness, indium content in QWs, and additionally to assess the structural quality of the samples.

## Results and discussion

### GaN:Si layers: unimplanted and implanted

#### Morphology

**Figure 3:**
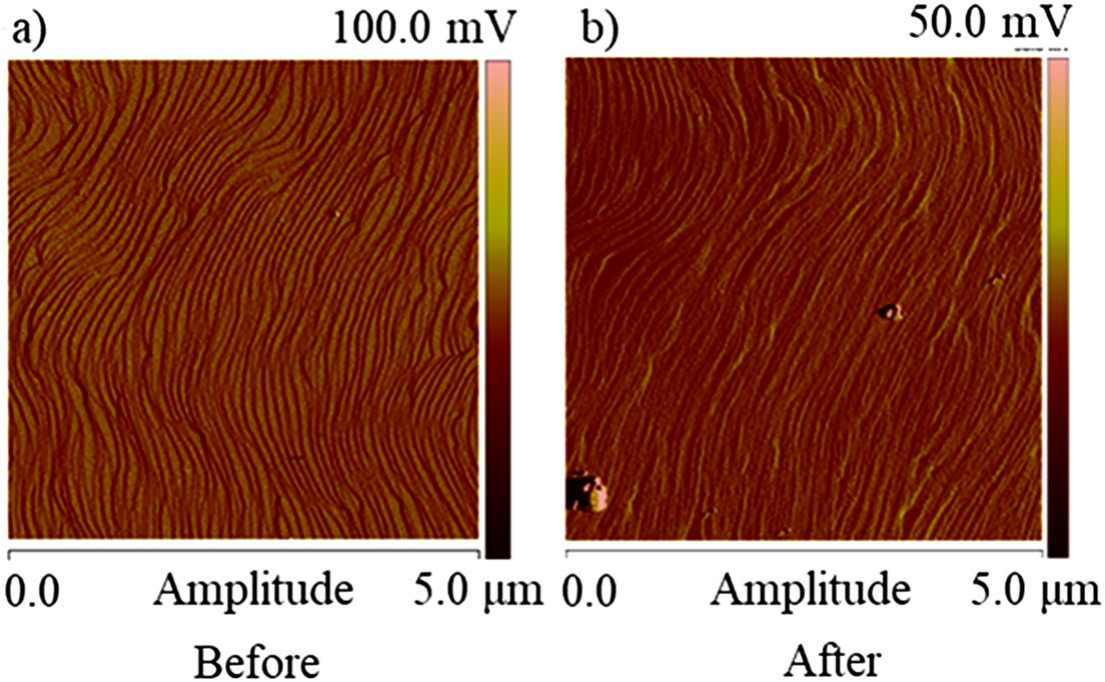
AFM images of the GaN:Si surface (**a**) before and (**b**) after He-ion implanatation. 20 nm SiN cap layer, was removed before AFM scans were made.

To be sure that InGaN/GaN MQWs would grow on substrates of the same morphology AFM scans were made. Comparison of the surface morphology before and after implantation is presented in Fig. 3a,b. Figure 3a shows an AFM scan of the surface of the unimplanted GaN:Si layer. It has revealed terraced structure with atomic layer steps. Similar AFM scan after ion implantation and removing of the SiN cap layer, is shown in Fig. 3b. Comparison of both scans shows that the SiN cap layer preserves the low surface roughness and atomic steps are still visible after ion implantation.

#### Structural characterization

**Figure 4:**
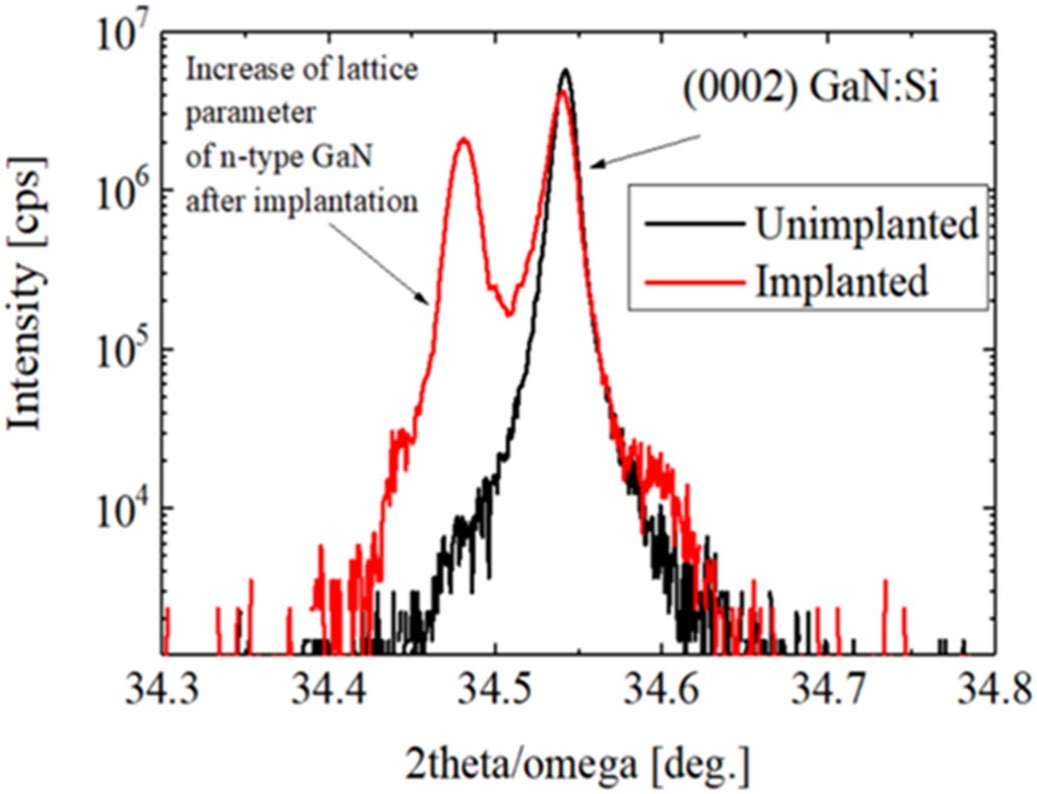
Comparison of the HRXRD 2theta/omega scans for the GaN:Si layers prior (black line) and after (red line) He-ion implanatation.

Structural changes in the GaN:Si layer caused by ion bombardment were examined by HRXRD and STEM. Comparison of 2theta/omega scans of the layers prior and after ion implantation is shown in Fig. 4. As a result of ion implantation, a peak at lower angle with respect to GaN (0002) appeared. Such an effect was observed also by other authors for GaN implantated with Mg, Ar, Be or Ca ions^19–22^. At low ion fluences it is attributed to GaN lattice expansion caused by displacement of atoms and/or creation of small dislocation loops. The estimated change of the lattice parameter c is Δc/c = 0.2%.

**Figure 5:**
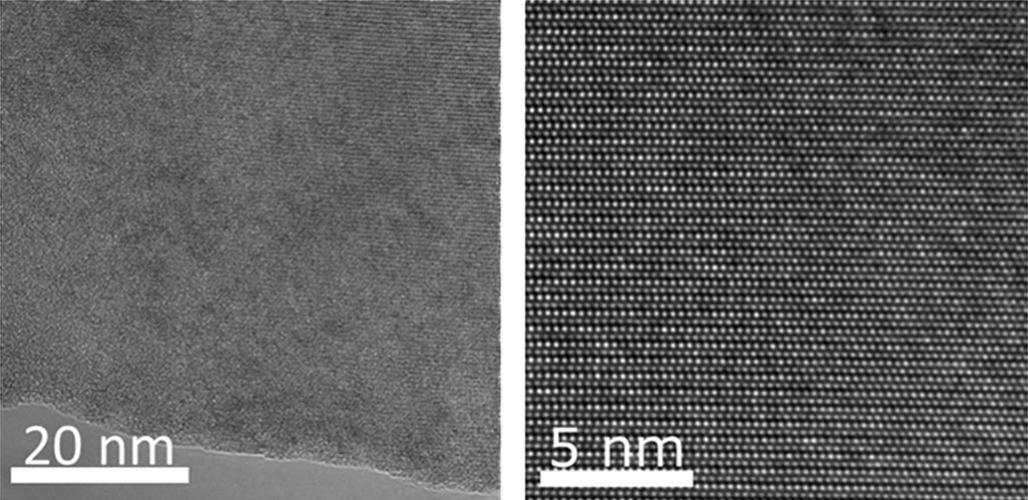
STEM images of GaN:Si layer after helium ions implantation with different scale. No extended defects was detected.

The ion fluence applied was too low to produce plastic deformation of the implanted layer^20,23^ what was confirmed by STEM measurements as shown in Fig. 5. Additionally STEM images show that the He ions at energies and fluences used do not produce extended defects—the implanted region is still crystalline with a well-defined atomic ordering.

#### Optical characterization

**Figure 6:**
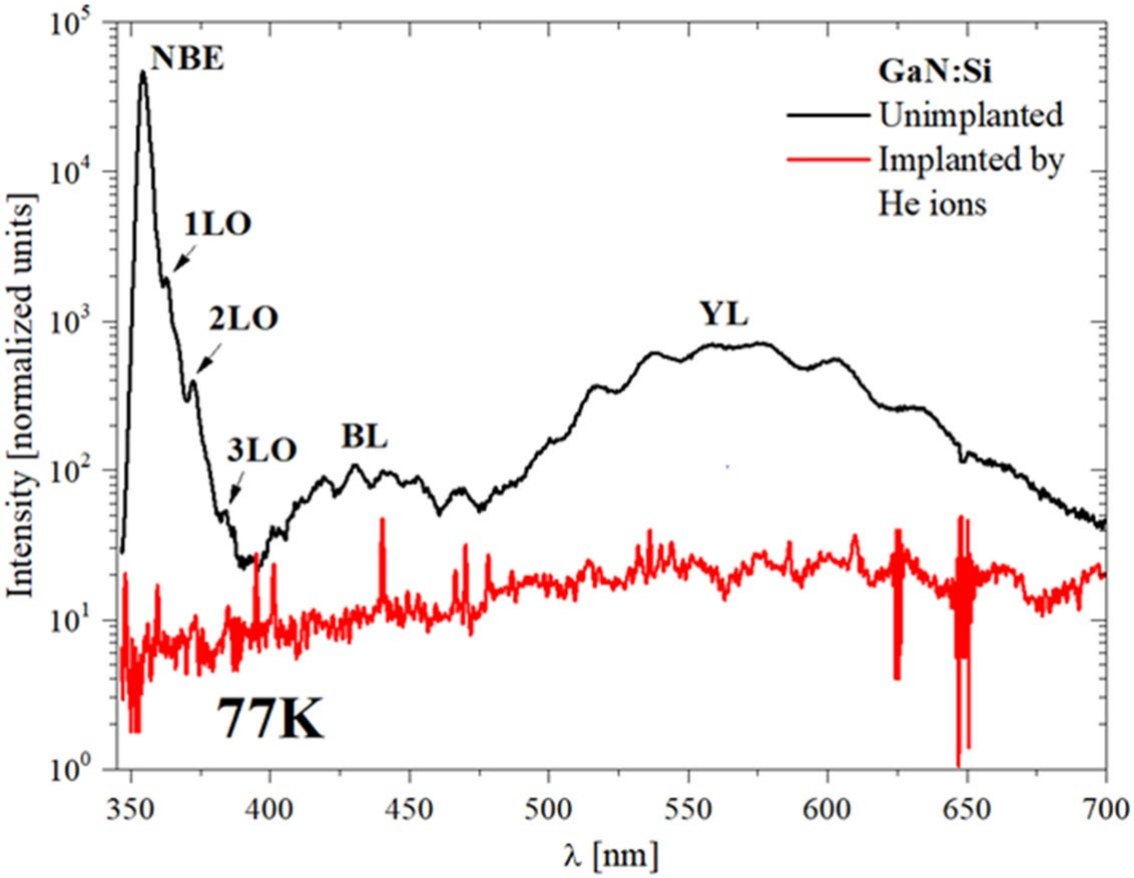
Comparison of low temperature (77 K) PL spectra of the GaN:Si layer prior (black line) and after (red line) He-ion implanation. Regions of the spectra corresponding to near-band emission (NBE), phonon replicas (LO), blue luminescence (BL) and yellow luminescence (YL) are marked on the graph. PL intensity was normalized to exposure time 100 ms.

The next step was to examine the impact of implantation on PL. Comparison of the PL spectra for implanted and unimplanted GaN:Si layers is presented in Fig. 6. In the case of unimplanted layer, standard near-band emission (NBE), phonon replicas (LO), blue luminescence (BL) and yellow luminescence (YL) are visible. In the case of layer after implantation, the PL signal was quenched in the entire spectrum. This effect is attributed to the creation of points defects, e.g., V_Ga_, V_N_, Ga_i_ or N_i_, which act as non-radiative recombination centers^11,12,17,24^.

### InGaN/GaN MQWs grown on unimplanted and implanted GaN:Si layers

#### Morphology

**Figure 7:**
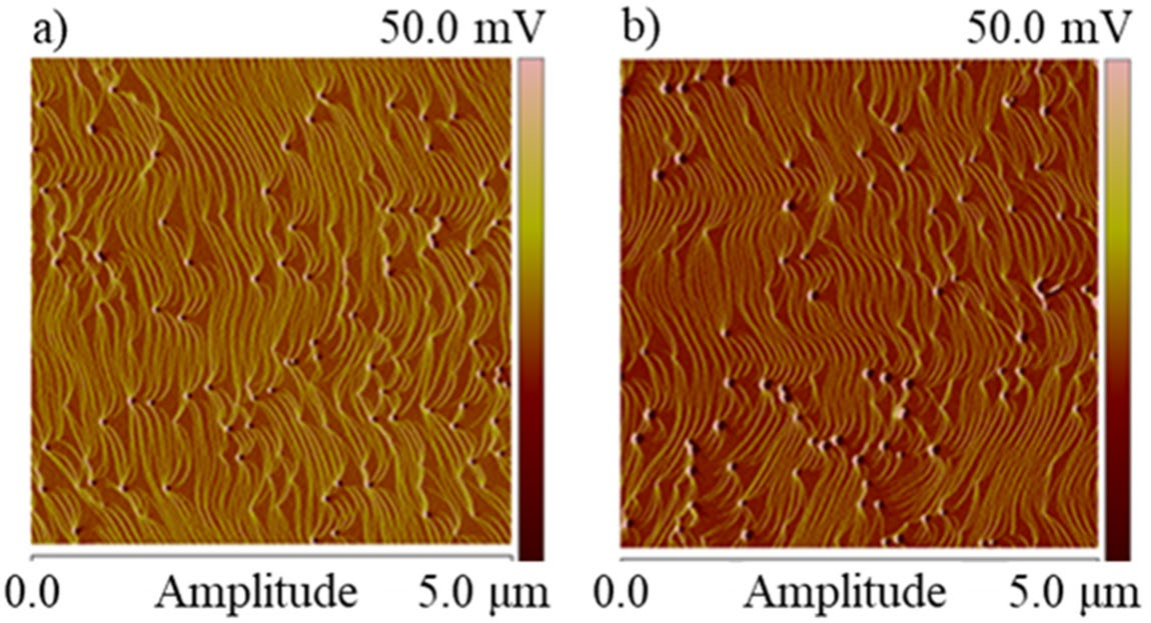
AFM images of InGaN/GaN MQWs structure grown on the unimplanted (**a**) and implanted (**b**) GaN:Si on the GaN:Si/sapphire template.

The surface morphology of the MQWs grown on unimplanted and implanted GaN:Si layers was studied using AFM. Figure 7 shows AFM scans of the MQWs surfaces grown on the unimplanted (Fig. 7a) and the implanted layers (Fig. 7b). In both cases, atomic steps and similar amount of “V-pits” (typical for InGaN^25^) are clearly visible.

#### Structural characterization of InGaN/GaN MQWs

**Figure 8:**
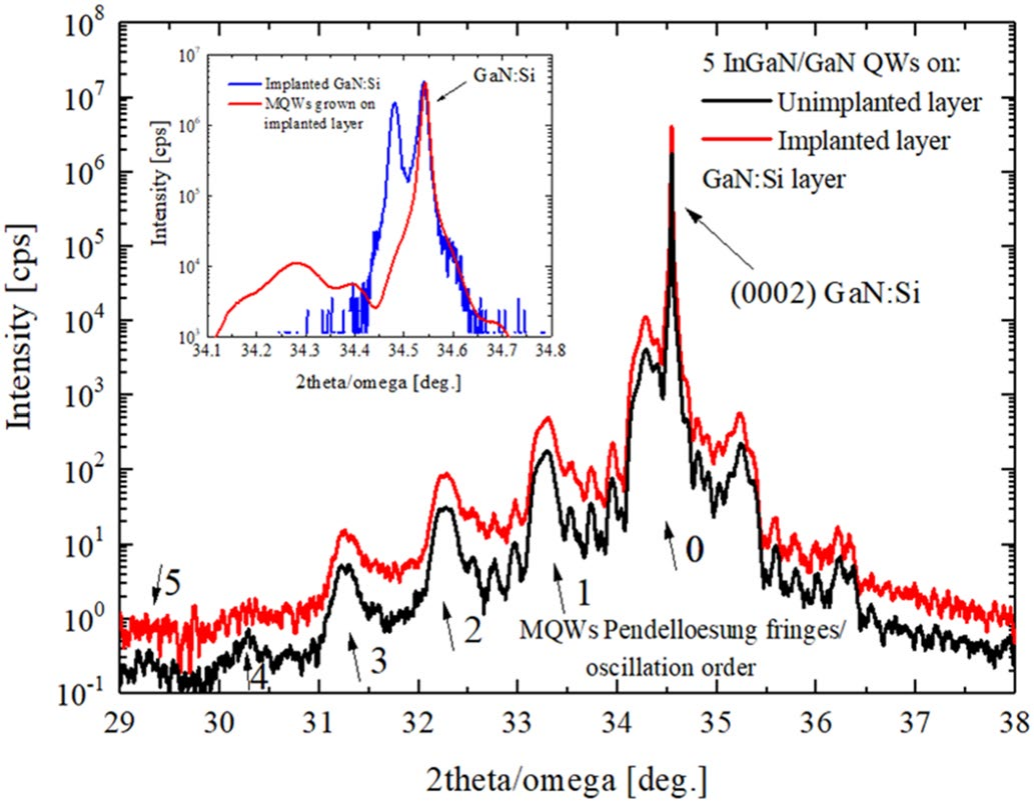
Comparison of HRXRD 2theta/omega scans of the MQWs grown on unimplanted (black line) and implanted GaN:Si layer (red line). Insert: comparison of the diffraction pattern of implanted GaN:Si (blue line) to the same layer with MQWs grown on it.

A set of HRXRD 2theta/omega scans of (0002) GaN reflection of both grown structures is shown in Fig. 8. Based on measured HRXRD patterns, using Epitaxy software, simulations were performed to determine the thickness of the QBs and QWs and also the indium content in the QWs. The parameters obtained are given in Table 1.

**Table 1:** Calculated thickness of the QBs and QWs and also In content based on HRXRD data.

MQWs grown on GaN:Si layer:	QB width [nm] ± 0.1 nm	QW width [nm] ± 0.1 nm	In content [%] ± 1%
Unimplanted	6.7	2.4	19
Implanted	6.8	2.4	20

It can be stated that in both cases, the good quality MQWs were grown with similar thickness and also with similar In content. It is worth to emphasize that the additional peak at lower angle which appeared as result of ion implantation of GaN:Si layer (Fig. 4), disappeared after growth of the MQWs structure (see insert in Fig. 8). It means that strain due to the defects presence in implanted GaN has been removed during growth of the MQWs^19^. However, intensities of the higher order oscillations (4th and 5th) are lower in case of MQWs grown on implanted layer (Fig. 8), actually 5th order oscillation is not observed—it means that the crystallographic quality of the MQWs/QB_S_ grown on implanted GaN:Si is somewhat worse as compared to growth on unimplanted layer. It can be due to diffusion of point defects from implanted layer towards MQWs during their growth.

#### Optical characterization of InGaN/GaN MQWs

**Figure 9:**
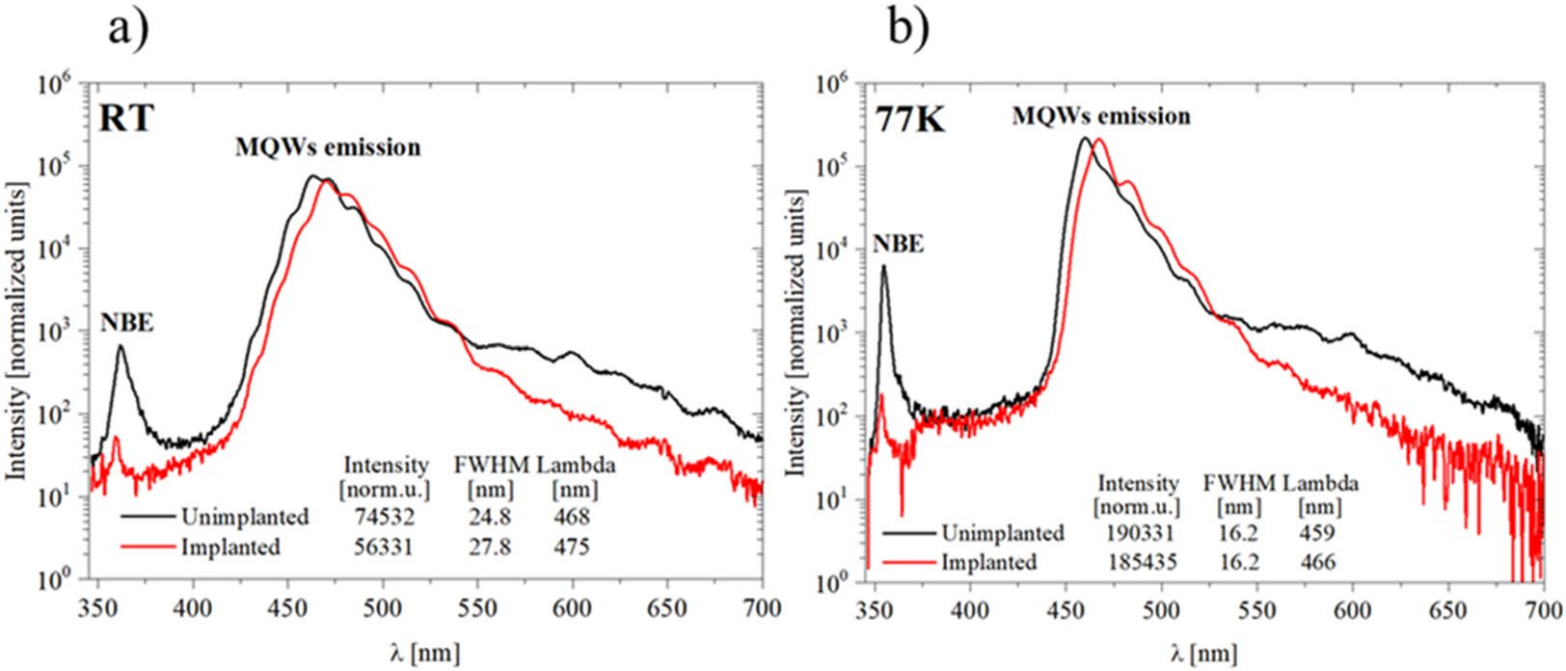
PL spectra collected at room temperature (**a**) and liquid nitrogen – 77 K, (**b**) of MQW structure grown on unimplanted (black line) and implanted GaN:Si (red line). PL intensity was normalized to exposure time 100 ms.

Optical properties of the MQWs structures were examined at RT and at 77 K and their comparison is shown in Fig. 9a and b. In the case of the MQWs grown on implanted layer, similarly to the implanted layer without MQWs, (Fig. 6), there is no peak for NBE of GaN both at RT and 77 K. It indicates that the strain removal observed in the HRXRD measurements was not accompanied by the full recovery of the implantation-induced point defects during MQWs growth.

Fitting parameters of the Gaussian function to PL spectra are presented in Fig. 9. At RT (Fig. 9a) MQWs grown on the implanted layer are characterized by a red shift (475 nm vs 468 nm) and about 25% lower intensity of MQWs emission and also higher FWHM, in relation to the structure grown on the unimplanted layer. Detected red shift can be caused by little bit wider QWs and little bit higher indium content in case of MQWs grown on implanted GaN:Si layer (see Table 1). Lower intensity and higher FWHM can be caused by diffusion of point defects to the QWs from the implanted layer below. These point defects start to act as non-radiative recombination centers and in consequence PL intensity decrease. PL measurements at 77 K show (Fig. 9b) that emission intensities of MQWs grown on unimplanted and implanted layers are similar (difference is only of 2.5% compared to 25% at RT) because at 77 K non-radiative recombination centers are almost “switched off”. Additionally, in both cases, FWHM are exactly the same, what strongly indicates that there is no more difference between these two samples, besides amount of point defects.

### Decomposition of InGaN/GaN MQWs caused by thermal stress

#### Structural characterization of InGaN/GaN MQWs after annealing

Structural characterization of the MQWs grown on unimplanted and implanted GaN:Si layers and annealed at 900 °C, 920 °C and 940 °C was carried out using HRXRD and STEM methods. The HRXRD measurement reveal averaged information from the whole area (1 mm × 10 mm) of the sample examined, while STEM it is a local type of analysis.

**Figure 10:**
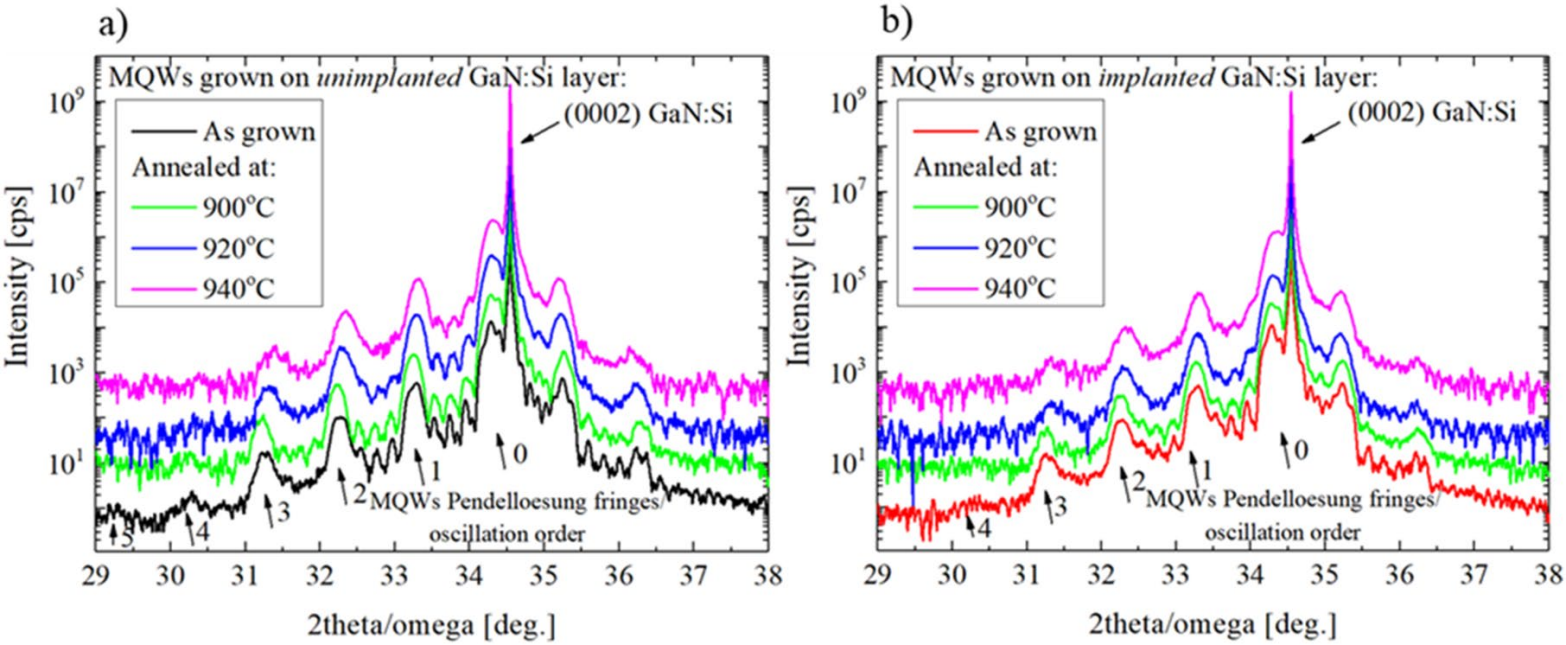
HRXRD 2theta/omega scans for the MQWs grown on unimplanted (**a**) and implanted (**b**) GaN:Si layer and after annealing at 900 °C, 920 °C and 940 °C.

Figures 10a and b show sets of 2theta/omega scans for InGaN/GaN MQWs before and after annealing, grown on unimplanted and implanted GaN:Si layers. It can be seen that annealing-induced changes of the diffraction patterns are faster in case of the MQWs grown on the implanted GaN layer: intensities of the Pendeloesung fringes decrease and FWHM increase faster in comparison to the MQWs grown on unimplanted layer.

**Figure 11:**
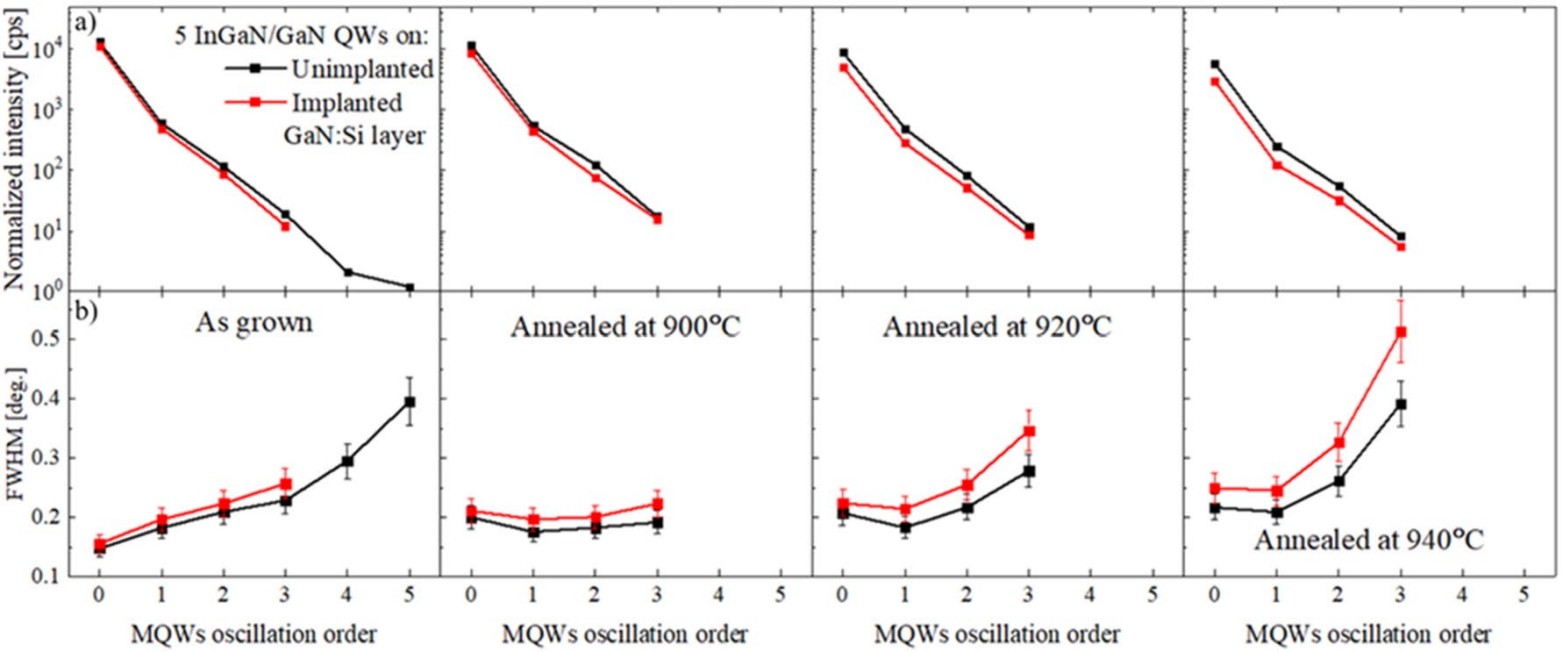
HRXRD data analysis of normalized intensity (**a**) (error at the level of 5% of the value) and FWHM (**b**) (error at the level of 10% of the value) of the Pendeloesung frings of MQWs grown on the unimplanted and implanted GaN:Si and after annealing at 900 °C, 920 °C and 940 °C.

Detailed analysis of the intensities and FWHM of the Pendeloesung fringes is shown in Fig. 11a and b. Intensities data (Fig. 11a) were normalized to the intensity of GaN (0002) reflection. Initial small differences in intensities and FWHM between as grown samples (Fig. 11a and b—first column) started to increase after annealing at 900 °C (Fig. 11a and b—second column). In case of MQWs grown on unimplanted layer, Pendelloesung fringes 5^th^ order disappear and 4^th^ order were too weak to be analyzed. For the implanted sample, the intensity of the lower order peaks decrease and simultaneously FWHM increase compared to MQWs grown on unimplanted layer. It can be concluded from XRD data that some structural changes take place after annealing at 900 °C.

**Figure 12:**
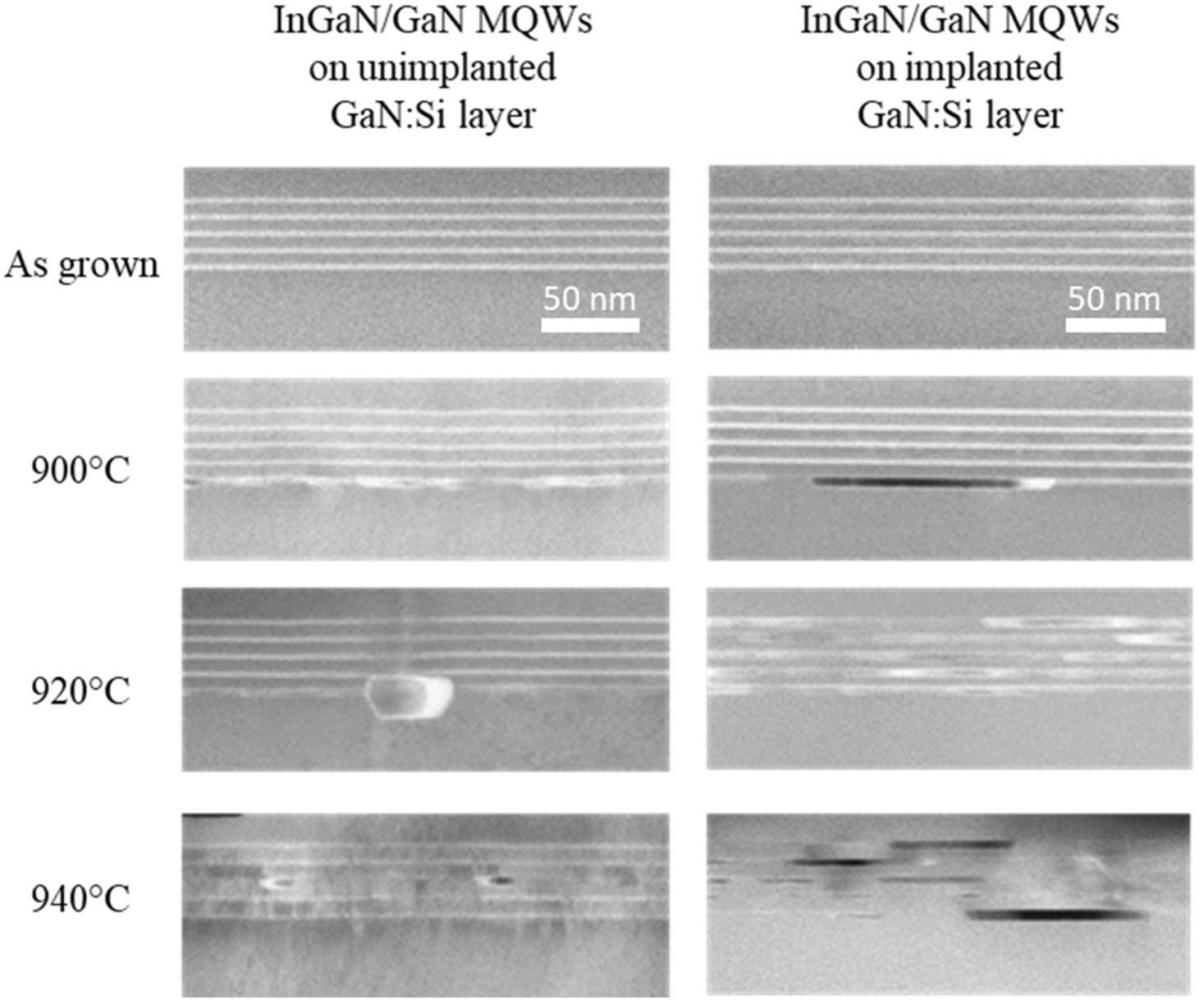
Set of STEM images of MQWs grown on unimplanted and implantes GaN:Si layer, as grown and after annealing at 900 °C, 920 °C and 940 °C. All images have the same scale.

STEM pictures (Fig. 12—second row) reveal that the decomposition of the first QW after annealing at 900 °C in both cases can be observed. Trapezoid-shape objects appear in the first grown QW. These objects consist of the metallic In precipitation and voids. Around such voids an InGaN shell with a very high In content of about 80% is formed (calculated by tetragonal distortion method). STEM pictures also indicate that larger voids appear in the first QW grown on the implanted GaN layer what is consistent with the XRD data (more pronounced changes, especially of FWHM of Pendelloesung fringes in that case shown in Fig. 11b—second column).

Significantly larger differences between both structures are observed after annealing at 920 °C. In both cases, i.e., for the InGaN/GaN MQWs grown on unimplanted and implanted layers, HRXRD pattern (Fig. 10a and b—blue lines) revealed further changes as compared to the as-grown samples (Fig. 10a and b—black line). Detailed analysis of the intensities and FWHM (Fig. 11a and b—third column) indicates that more pronounced structural changes took place in the MQWs grown on the implanted layer: intensities decrease and FWHM of Pendeloesung fringes increase compared to the MQWs grown on the unimplanted layer. In a series of STEM pictures (Fig. 12—third row), we observed that all five QWs (70% of specimen) or four QWs (30% of specimen) got decomposed in the case of MQWs grown on implanted layer in contrast to just one decomposed QW on the unimplanted layer as a result of annealing at 920˚C.

Further changes of diffraction patterns can be observed (Fig. 10a and b—magenta line) after annealing at 940 °C—it suggest further structural changes of the MQWs in both cases but more pronounced in case of the MQWs grown on the implanted GaN:Si layer. Comparison of the detailed analysis of the Pendeloesung fringes for both samples (Fig. 11a and b—fourth column) shows that after annealing at 940 °C, differences in intensities and FWHM values increase. It suggests widening difference in the decomposition stage of the MQWs grown on both types of GaN:Si layer. STEM images (Fig. 12—last row) confirmed that further increase of annealing temperature up to 940˚C led to a full decomposition of all 5 QWs (100% of specimen) grown on the implanted GaN:Si layer. In the case of the sample on unimplanted GaN:Si layer, we observe an enlargement of the voids as compared to the lower annealing temperature. In that case, the first, second or third QW got decomposed, but never all at the same time (in Fig. 12 we observe decomposed third QW only).

#### Optical characterization of InGaN/GaN MQWs after annealing

**Figure 13:**
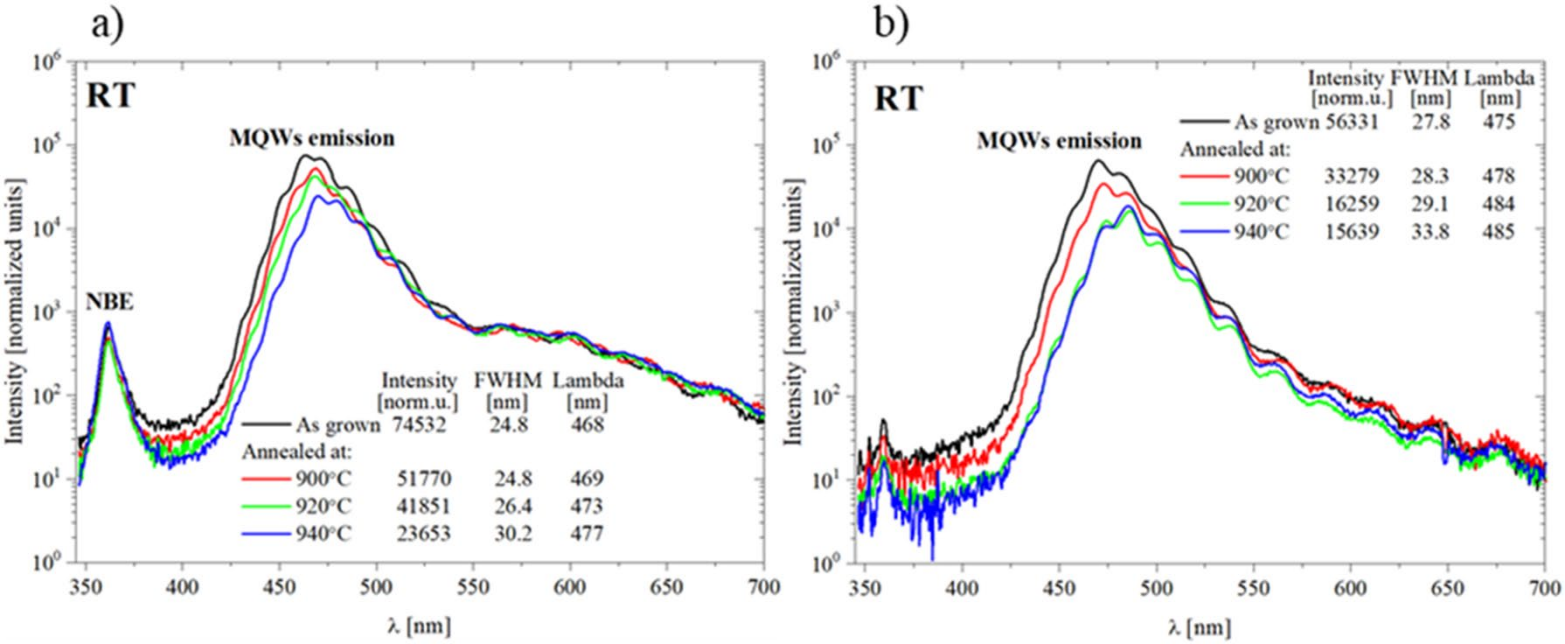
Room temperature spectra of the MQW structure: (**a**) grown on unimplanted and (**b**) on implanted GaN:Si. Black lines: as grown samples. Red lines: after annealing at 900 °C. Green lines: after annealing 920 °C. Blue lines: after annealing at 940 °C. PL intensity was normalized to exposure time 100 ms.

**Figure 14:**
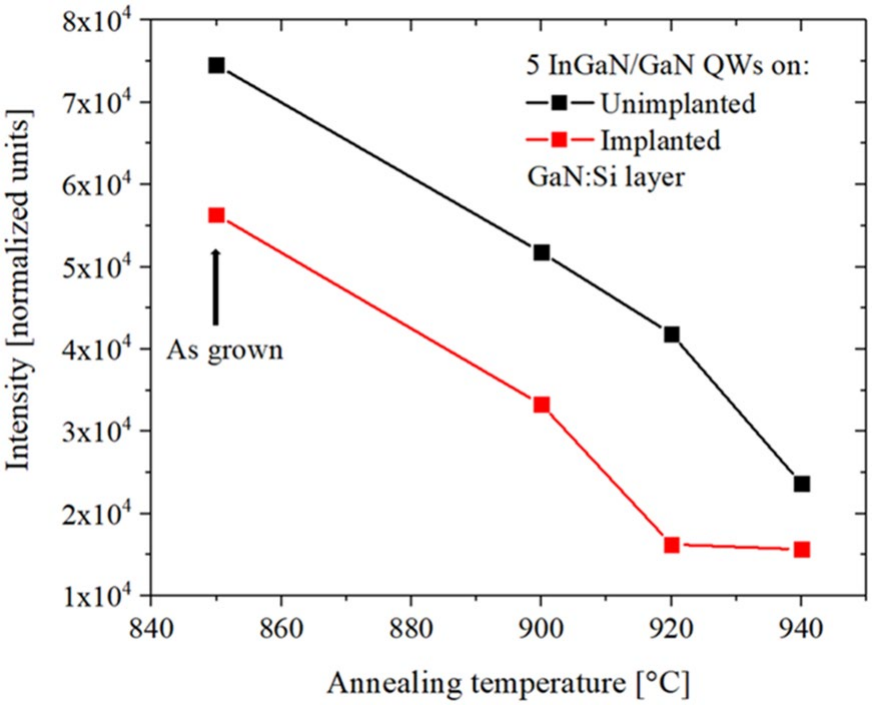
Intensity of the PL spectra of the MQW structure grown on unimplanted (black line) and implanted GaN:Si (red line) as grown (850 °C) and after annealing (900 °C, 920 °C, 940 °C). PL intensity was normalized to exposure time 100 ms.

Optical properties of the MQWs structures grown on the unimplanted and implanted GaN:Si layers after annealing at 900 °C, 920 °C and 940 °C were examined at RT and the PL spectra are shown in Fig. 13a and b. It can be observed that annealing of the samples resulted in decreasing intensities and increasing FWHM of the emitted spectra in both cases. Detailed analysis of the measured intensities in both annealed samples is shown in Fig. 14. In case of MQWs grown on unimplanted GaN:Si layer changes of the intensities up to 920 °C are almost linear (it corresponds to one decomposed QW and enlargement of the voids) and after annealing at 940 °C decrease of the measured intensity is larger (it corresponds to decomposed different QWs in the structure, but never simultaneously). In the case of MQWs grown on implanted layer, after annealing at 900 °C change of the intensity is similar to that observed for unimplanted layer (it correspond to one decomposed QW), then after annealing at 920 °C the change is much more pronounced (it correspond to 4–5 decomposed QWs). Annealing at 940 °C results in small changes of the intensities (all 5 decomposed QWs and enlargement of the voids). In conclusion, the optical changes follow the structural changes in the samples, i.e., their stages of the decomposition.

## Conclusions

Complementary analytical techniques were applied to study the role of point defects migrating at elevated temperatures from the GaN:Si layers on the decomposition of the InGaN QWs grown above. Point defects were introduced into the GaN:Si layer using He-ion implantation. Photoluminescence optical studies indicate that these defects were not fully removed during MQWs growth on implanted layer.

The InGaN/GaN MQWs grown on the implanted GaN:Si are of almost the same crystallographic quality as those grown on the unimplanted GaN:Si, but two observations indicate that they contain point defects diffused from the implanted region: (i) PL spectra parameters are slightly worse, (ii) intensities of the higher order Pendeloesung fringes in XRD are lower.

Structural studies (HRXRD and STEM results) have shown that the thermal decomposition of InGaN/GaN QWs begins from the lowest well after annealing at 900 °C in both cases. After annealing at 920 °C, HRXRD and STEM results indicate that in the case of the MQWs grown on the implanted layer, rapid decomposition of almost all the QWs occurs, in contrast to the QWs grown on the unimplanted GaN layer for which only one quantum well was decomposed. After annealing at 940 °C, we observe larger voids and decomposed all QWs grown on the implanted GaN layer and still decomposed only one (first, second or third) QW grown on the unimplanted GaN layer.

The experimental data show that the migration of point defects introduced by He ion-implantation into the GaN:Si layer contributed to the decomposition of all quantum wells at a lower temperature than it was the case for the QWs grown on the unimplanted layer.

The most probable defects responsible for the InGaN QWs decomposition are gallium (and/or indium) vacancies V_Ga_ (or V_In_) and In_Ga_V_Ga_ clusters. Since such defects are present in GaN:Si and their energy barrier for migration is very low^26^, the decomposition begins from the first grown InGaN QW.

The issue of the diffusion of point defects into the QWs must be further explored. One can see that the diffusion rate must be significant as in our experiment there was 56.5 nm thick GaN layer between InGaN MQWs and the implanted region. The diffusion is enhanced by the presence of dislocations (high density of more than 10^8^ cm^−2^), by electric field and strain. These factors should be varied in further experiments.

An interesting additional issue is the point defect annihilation during the InGaN/GaN MQWs growth. Some of these defects are annihilated what can be seen in the HRXRD scan (the peak related to damaged region disappears), but some not, as the PL signal is not recovered.

## References

[1] Schulz S, Caro MA, Coughlan C, O'Reilly EP (2015). Atomistic analysis of the impact of alloy and well-width fluctuations on the electronic and optical properties of InGaN/GaN quantum wells. Phys. Rev. B..

[2] Hammersley S (2015). Effects of quantum well growth temperature on the recombination efficiency of InGaN/GaN multiple quantum wells that emit in the green and blue spectral regions. Appl. Phys. Lett..

[3] Holec D (2008). Equilibrium critical thickness for misfit dislocations in III-nitrides. J. Appl. Phys..

[4] Haller C (2017). Burying non-radiative defects in InGaN underlayer to increase InGaN/GaN quantum well efficiency. Appl. Phys. Lett..

[5] Haller C (2018). GaN surface as the source of non-radiative defects in InGaN/GaN quantum wells. Appl. Phys. Lett..

[6] Queren D (2009). Quality and thermal stability of thin InGaN films. J. Cryst. Growth.

[7] Oh M-S (2006). Improvement of green LED by growing p-GaN on In0.25GaN/GaN MQWs at low temperature. J. Cryst. Growth.

[8] Van Daele B, Van Tendeloo G, Jacobs K, Moerman I, Leys MR (2004). Formation of metallic In in InGaN/GaN multiquantum wells. Appl. Phys. Lett..

[9] Saarinen K (1997). Observation of native Ga vacancies in GaN by positron annihilation. Phys. Rev. Lett..

[10] Tuomisto F, Pelli A, Yu KM, Walukiewicz W, Schaff WJ (2007). Compensating point defects in ^4^He^+^-irradiated InN. Phys. Rev. B.

[11] Kucheyev SO, Williams JS, Pearton SJ (2001). Ion Implantation into GaN. Mater. Sci. Eng. Rep..

[12] Ronning C, Carlson EP, Davis RF (2001). Ion implantation into gallium nitride. Phys. Rep..

[13] Kucheyev SO, Williams JS, Jagadish C, Zou J, Li G (2000). Damage buildup in GaN under ion bombardment. Phys. Rev. B.

[14] Wenzel A, Liu C, Rauschenbach B (1999). Effect of implantation-parameters on the structural properties of Mg-ion implanted GaN. Mater. Sci. Eng. B.

[15] Prozheeva V (2017). Radiation-induced alloy rearrangement in In_x_Ga_1-x_N. Appl. Phys. Lett..

[16] Turos A (2013). On the mechanism of damage buildup in gallium nitride. Radiat. Eff. Defects Solids.

[17] Kucheyev SO (2001). Cathodoluminescence depth profiling of ion-implanted GaN. Appl. Phys. Lett..

[18] Ziegler JF, Ziegler MD, Biersack JP (2010). SRIM: The stopping and range of ions in matter. Nucl. Instrum. Methods Phys. Res., Sect. B.

[19] Chi GC, Pong BJ, Pan CJ, Teng YC (1997). Characterizations of Mg Implanted GaN. MRS Proc..

[20] Liu C, Mensching B, Volz K, Rauschenbach B (1997). Lattice expansion of Ca and Ar ion implanted GaN. Appl. Phys. Lett..

[21] Ronning C (1999). Characterization of Be-implanted GaN annealed at high temperatures. MRS Proc..

[22] Liu C, Schreck M, Wenzel A, Mensching B, Rauschenbach B (2000). Damage buildup and removal in Ca-ion-implanted GaN. Appl. Phys. A.

[23] Tan HH (1996). Damage to epitaxial GaN layers by silicon implantation. Appl. Phys. Lett..

[24] Langer T (2013). Nonradiative recombination due to point defects in GaInN/GaN quantum wells induced by Ar implantation. Proc. SPIE.

[25] Smalc-Koziorowska J, Grzanka E, Czernecki R, Schiavon D, Leszczynski M (2015). Elimination of trench defects and V-pits from InGaN/GaN structures. Appl. Phys. Lett..

[26] Hrytsak R, Kempisty P, Grzanka E, Leszczynski M, Sznajder M (2020). DFT study on point defects migration through the pseudomorphic and lattice-matched InN/GaN interfaces. Comput. Mater. Sci..

